# The Treatment Cascade for Chronic Hepatitis C Virus Infection in the United States: A Systematic Review and Meta-Analysis

**DOI:** 10.1371/journal.pone.0101554

**Published:** 2014-07-02

**Authors:** Baligh R. Yehia, Asher J. Schranz, Craig A. Umscheid, Vincent Lo Re

**Affiliations:** 1 Department of Medicine, University of Pennsylvania Perelman School of Medicine, Philadelphia, Pennsylvania, United States of America; 2 Leonard Davis Institute of Health Economics, University of Pennsylvania, Philadelphia, Pennsylvania, United States of America; 3 Center for Clinical Epidemiology and Biostatistics, Department of Biostatistics and Epidemiology, University of Pennsylvania Perelman School of Medicine, Philadelphia, Pennsylvania, United States of America; 4 Department of Medicine, New York University School of Medicine, New York, New York, United States of America; 5 Center for Evidenced-Based Practice, University of Pennsylvania Health System, Philadelphia, Pennsylvania, United States of America; Mayo Clinic, United States of America

## Abstract

**Background:**

Identifying gaps in care for people with chronic hepatitis C virus (HCV) infection is important to clinicians, public health officials, and federal agencies. The objective of this study was to systematically review the literature to provide estimates of the proportion of chronic HCV-infected persons in the United States (U.S.) completing each step along a proposed HCV treatment cascade: (1) infected with chronic HCV; (2) diagnosed and aware of their infection; (3) with access to outpatient care; (4) HCV RNA confirmed; (5) liver fibrosis staged by biopsy; (6) prescribed HCV treatment; and (7) achieved sustained virologic response (SVR).

**Methods:**

We searched MEDLINE, EMBASE, and the Cochrane Database of Systematic Reviews for articles published between January 2003 and July 2013. Two reviewers independently identified articles addressing each step in the cascade. Studies were excluded if they focused on specific populations, did not present original data, involved only a single site, were conducted outside of the U.S., or only included data collected prior to 2000.

**Results:**

9,581 articles were identified, 117 were retrieved for full text review, and 10 were included. Overall, 3.5 million people were estimated to have chronic HCV in the U.S. Fifty percent (95% CI 43–57%) were diagnosed and aware of their infection, 43% (CI 40–47%) had access to outpatient care, 27% (CI 27–28%) had HCV RNA confirmed, 17% (CI 16–17%) underwent liver fibrosis staging, 16% (CI 15–16%) were prescribed treatment, and 9% (CI 9–10%) achieved SVR.

**Conclusions:**

Continued efforts are needed to improve HCV care in the U.S. The proposed HCV treatment cascade provides a framework for evaluating the delivery of HCV care over time and within subgroups, and will be useful in monitoring the impact of new screening efforts and advances in antiviral therapy.

## Introduction

The HIV treatment cascade (diagnosis, linkage to care, retention in care, prescription of antiretroviral therapy, and viral suppression) is an effective tool for improving the health of people living with HIV (PLWH) and for achieving the public health benefits of antiretroviral therapy [1], [2]. It has been used by federal, state, and local agencies to identify gaps in the delivery of care, prioritize and target resources, and monitor the United States (U.S.) *National HIV/AIDS Strategy* [2]–[6]. As different patient behaviors and health system mechanisms are required to successfully complete each step of the cascade, it also demonstrates the need for coordinated action to meet treatment goals [7].

Similar to PLWH, people with chronic hepatitis C virus (HCV) infection need to fulfill several steps along a care continuum to achieve optimal health outcomes [8], [9]. First, individuals must be diagnosed and aware of their HCV infection and seek care [10]. Once in care, patients should have HCV RNA confirmation testing and undergo liver fibrosis staging to help inform prognosis and make decisions regarding HCV therapy [11]. Lastly, individuals must receive and adhere to HCV treatment to achieve a sustained virologic response (SVR) [12]. Throughout this continuum, patients should receive recommended screenings, vaccinations, and care as outlined by national guidelines [11], [13].

This HCV treatment cascade aligns with the goals of the U.S. *Action Plan for the Prevention, Care, and Treatment of Viral Hepatitis*, which established early identification, linkage to care and treatment, and improved quality of care as top priorities for combating the silent epidemic of chronic HCV infection [14]. As antiviral therapy for chronic HCV advances to become more convenient, effective, and better-tolerated, monitoring the HCV treatment cascade will become increasingly important to clinicians, public health officials, and federal agencies [15]. This systematic review and meta-analysis builds on prior studies evaluating rates of HCV detection, referral to care, and treatment [16], [17], and integrates published data to provide estimates of the number of chronic HCV-infected persons living in the U.S. completing each step along the HCV treatment cascade. These data will help identify key deficits in chronic HCV care that will be important for the development of programs to improve diagnosis, linkage to care, and management of this disease.

## Methods

### Data Sources and Searches

We examined data addressing 7 key steps along the HCV treatment cascade to identify the number of people with: (1) chronic HCV infection; and the proportion of chronic HCV-infected individuals: (2) diagnosed and aware of their infection; (3) with access to outpatient care; (4) HCV RNA confirmed; (5) disease staged by liver biopsy; (6) prescribed HCV treatment; and (7) achieving SVR [16], [17]. We searched MEDLINE, EMBASE, and the Cochrane Database of Systematic Reviews for English language articles published between January 2003 and July 2013 using keywords and structured language representing each step along the HCV treatment cascade [18]. Details appear in **Table S1**.

### Study Selection

Titles and abstracts were screened by a single reviewer (BRY or AS). Articles were selected for full-text review if they were relevant to HCV treatment cascade steps. Two independent reviewers (BRY and VLR) evaluated articles selected for full-text review using a standardized form. Disagreements were resolved by consensus.

Studies were included if they addressed one or more steps along the HCV treatment cascade, were conducted inside the U.S., presented original data, and used data collected after January 1, 2000. We intentionally focus on studies conducted inside the U.S., and not in other developed counties, as multiple steps in the treatment cascade are directly influenced by the healthcare delivery system and these systems vary widely across countries [19]. The use of data collected after January 1, 2000 corresponds with the introduction of dual therapy with a pegylated interferon and ribavirin for the treatment of chronic HCV infection that occurred in the early 2000 s, and was also selected to ensure the use of the most current data available. To improve generalizability, single site studies and those exclusively focusing on specific populations (e.g., only immigrants, injection drug users, those with HIV/HCV co-infection) were excluded. If multiple publications resulted from the same patient sample or longitudinal cohort, we selected the most inclusive and current study. Bibliographies of included studies were reviewed for additional studies, and three experts in chronic HCV infection were contacted to ensure no relevant studies were missed.

### Data Extraction

Data from each included study were extracted by one author (BRY) into tables stratified by HCV treatment cascade step and verified by another author (VLR) for accuracy. Discrepancies were resolved through consensus. We extracted information on study design, period, population, sample size, definition of outcome measure(s), and raw data to permit calculation of estimates of chronic HCV-infected persons completing each cascade step.

### Data Synthesis and Analysis

The number of chronic HCV-infected persons completing each step along the HCV treatment cascade was determined. After ascertaining the initial estimate of chronic HCV-infected persons in the U.S. (cascade step 1), we estimated the number of chronic HCV-infected persons diagnosed and aware of their infection (cascade step 2). Among those diagnosed and aware of their chronic HCV infection, we estimated the number with access to outpatient care (cascade step 3). Next, among chronic HCV-infected persons diagnosed and aware of their infection and with access to outpatient care, we estimated the number who had: HCV RNA confirmed (cascade step 4), underwent a liver biopsy (cascade step 5), and antiviral therapy prescribed (cascade step 6). Since cascade steps 4–6 had the same denominator, the proportion of persons prescribed HCV treatment was not conditioned on the number with HCV RNA confirmation and liver biopsy. Finally, among persons prescribed HCV treatment, we estimated the number that achieved a sustained virologic response (cascade step 7). Using the above estimates, we calculated the proportion of chronic HCV-infected persons completing each step along the HCV treatment cascade by dividing the number of people who completed each step (numerator) by the number of chronic HCV-infected persons in the U.S. (denominator, obtained from cascade step 1).

Since multiple studies were identified for HCV treatment cascade steps 5–7, we performed random-effects meta-analyses for these steps to determine pooled prevalence estimates with 95% confidence intervals (CIs). We assessed the heterogeneity between study results using the I^2^ statistic. Significant heterogeneity was defined as an I^2^ statistic ≥50% [20]. Analyses were performed in OpenMeta[Analyst] [21].

The U.S. Veterans Health Administration (VA) is the largest single provider of HCV care in the U.S., currently caring for approximately 165,000 veterans with chronic HCV infection [13]. Because of differences in the prevalence of comorbid diseases, as well as the management and treatment of chronic HCV infection between U.S. Veterans and non-Veterans [13], we distinguished VA and non-VA studies. The primary analysis focused on non-VA studies. In a secondary analysis, we preferentially selected VA studies when available.

## Results

**Figure 1:**
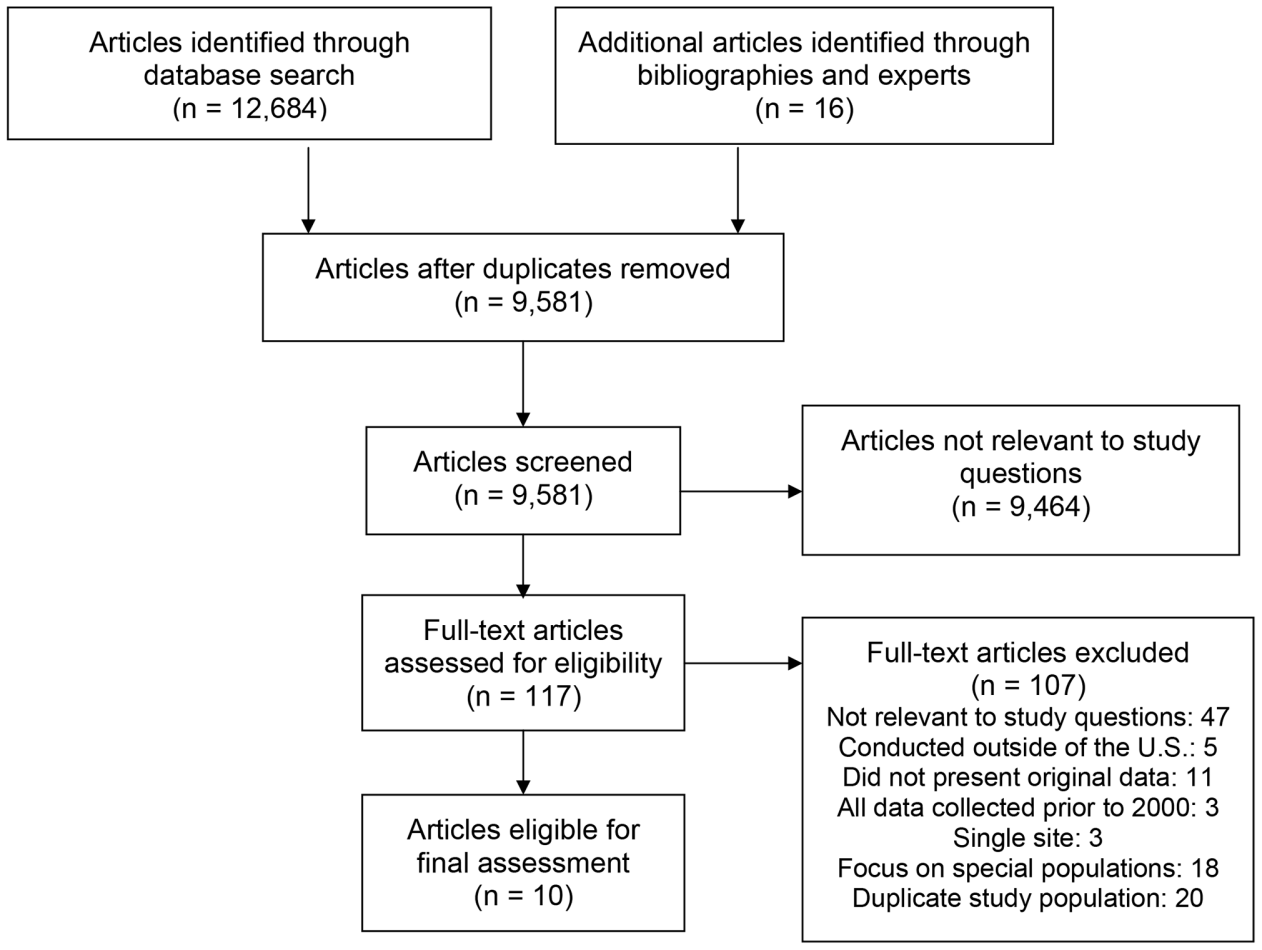
Summary of Article Search, Screening, and Selection Process.

Our literature search yielded 9,581 citations (excluding duplicates), of which 117 met eligibility for full text review (**Figure 1**). Of those, 107 were excluded because they did not address any of the study questions, were conducted outside of the U.S., did not present original data, only analyzed data collected prior to 2000, involved a single site, focused on special populations, or examined study subjects used in a more recent study. Among the remaining 10 articles [22]–[31], three focused exclusively on U.S. Veterans. Details on the study populations, periods, and sample sizes of included studies by cascade step are shown in **Table 1**.

**Table 1:** Evaluation of chronic hepatitis C virus treatment cascade studies eligible for final assessment.

Author, Year	Data Source	Time Period	Sample Size	Estimate (95% CI)	Pooled Prevalence Estimate (95% CI)*
**Study Question 1: Number of people with chronic HCV infection**					
Armstrong et al, 2006	NHAS	1999–2002	15,079	3,500,000 people	–
**Study Question 2: Proportion aware of their infection**					
Younossi et al, 2013	NHAS	2001–2010	203	49.8% (42.9–56.7)	–
**Study Question 3: Proportion with access to healthcare, among those aware of their infection**					
Younossi et al, 2013	NHAS	2001–2010	101	86.9% (80.3–93.5)	–
**Study Question 4: Proportion HCV RNA confirmed, among those in care**					
Moorman et al, 2013	4 HCOs	2001–2010	8,810	62.9% (61.9–63.9)	–
**Study Question 5: Proportion disease staged by liver biopsy, among those in care**					
Moorman et al, 2013	4 HCOs	2001–2010	8,810	38.4% (37.4–39.4)	–
*Groessl et a, 2012*	*VA*	*1996–2006*	*171,893*	*16.7% (16.5–16.9)*	
**Study Question 6: Proportion prescribed HCV treatment, among those in care**					
Moorman et al, 2013	4 HCOs	2001–2010	8,810	36.4% (35.4–37.4)	36.7% (35.8–37.6)
Kanwal et al, 2010	Insurance claims	2003–2006	2,893	37.5% (35.7–39.3)	
*Kanwal et al, 2012*	*VA*	*2003–2006*	*34,749*	*17.9% (17.5–18.3)*	–
**Study Question 7: Proportion achieving SVR, among those in care and prescribed HCV treatment**					
Mitra et al, 2010	Insurance claims	2002–2006	575	58.4% (54.4–62.4)	58.8% (56.1–61.5)
Arora et al, 2011	22 clinics	2004–2009	407	58.0% (53.2–62.8)	
Russell et al, 2012	Health system	2002–2008	259	60.1% (54.1–66.1)	
*Backus et al, 2011*	*VA*	*2001–2009*	*16,864*	*44.1% (43.4–44.9)*	–

Abbreviations: HCOs = healthcare organization; NHANES = National Health and Nutrition Examination Survey; VA = Veterans Administration.

*Obtained from meta-analyses. These estimates do not include Veterans Administration-based studies (*in italics*).

**Figure 2:**
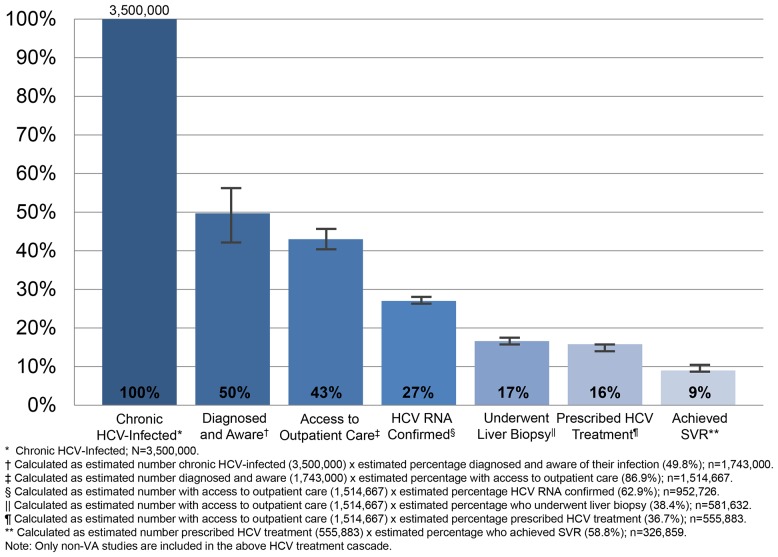
Treatment Cascade for People with Chronic Hepatitis C Virus (HCV) Infection, Prevalence Estimates with 95% Confidence Intervals. * Chronic HCV-Infected; N = 3,500,000. † Calculated as estimated number chronic HCV-infected (3,500,000) x estimated percentage diagnosed and aware of their infection (49.8%); n = 1,743,000. ‡ Calculated as estimated number diagnosed and aware (1,743,000) x estimated percentage with access to outpatient care (86.9%); n = 1,514,667. § Calculated as estimated number with access to outpatient care (1,514,667) x estimated percentage HCV RNA confirmed (62.9%); n = 952,726. || Calculated as estimated number with access to outpatient care (1,514,667) x estimated percentage who underwent liver biopsy (38.4%); n = 581,632. ¶ Calculated as estimated number with access to outpatient care (1,514,667) x estimated percentage prescribed HCV treatment (36.7%); n = 555,883. ** Calculated as estimated number prescribed HCV treatment (555,883) x estimated percentage who achieved SVR (58.8%); n = 326,859. Note: Only non-VA studies are included in the above HCV treatment cascade.

Based on National Health and Nutrition Examination Survey (NHANES) data from 1999 through 2002, approximately 3.2 million people in the U.S. have chronic HCV infection [22]. Since NHANES did not sample high-risk groups, particularly incarcerated, homeless, or institutionalized individuals, the actual prevalence of chronic HCV infection was estimated to be 3.5 million [22]. This estimate was used for cascade step 1 (**Figure 2**).

Among those living with chronic HCV, approximately 50% are unaware of their diagnosis based on data from the NHANES Hepatitis C Follow-Up Survey [31], [32]. A total of 500 participants who tested positive for HCV RNA between 2001 and 2010 were asked if they were aware of their HCV status before being notified by NHANES. Half of the 203 respondents were unaware of their HCV status [31], [32]. Thus, 1,743,000 chronic HCV-infected persons (49.8% [95% CI, 42.9–56.7%] of 3.5 million) are estimated to be diagnosed and aware of their infection (cascade step 2).

In this same study, participants who were aware of their chronic HCV status were also asked about their health insurance status [31], [32]. Among those aware of their chronic HCV infection, 86.9% had health insurance compared to 60.4% of those unaware of their HCV infection. Thus, 1,514,667 persons (43.3% [95% CI, 39.9–46.6%] of 3.5 million) are estimated to be aware of their chronic HCV diagnosis and have access to outpatient healthcare (cascade step 3).

Once in care, measurement of HCV RNA is needed to confirm diagnosis of chronic HCV infection, and staging of liver disease is important for guiding HCV treatment decisions. Data from the Chronic Hepatitis B and C Cohort Study, which included 8,810 patients with chronic HCV receiving care at four health systems in the U.S. (Detroit, Michigan; Danville, Pennsylvania; Honolulu, Hawaii; and Portland, Oregon), reported that 5,540 (62.9%) of these patients had confirmatory HCV RNA testing, and 3,380 (38.4%) had hepatic fibrosis staging with a liver biopsy during 2001–2010 [29]. Based on these data, 952,726 persons (27.2% [95% CI, 26.8–27.7%] of 3.5 million) are estimated to be aware of their chronic HCV diagnosis, have access to healthcare, and received confirmatory HCV RNA testing (cascade step 4) and 581,632 (16.6% [95% CI, 16.2–17.1%] of 3.5 million) are estimated to be aware of their chronic HCV diagnosis, have access to healthcare, and have underwent a liver biopsy for hepatic fibrosis staging (cascade step 5). In contrast, an evaluation of the national VA Hepatitis C Clinical Case Registry from 1997 to 2006 indicated that only 16.7% (28,677 of 171,893) of VA patients with chronic HCV received a liver biopsy [25]. Using these VA-based data, 252,949 persons (7.2% [95% CI, 7.1–7.3%] of 3.5 million) are estimated to be aware of their chronic HCV diagnosis, have access to healthcare, and received a liver biopsy (cascade step 5).

Two non-VA studies evaluated prescription of pegylated interferon plus ribavirin treatment among patients with chronic HCV infection [27], [29]. Given the recent introduction of direct-acting antiviral agents, we did not identify any population-based studies evaluating these agents that met our selection criteria. Kanwal and colleagues examined prescription rates among 2,893 patients enrolled in one of the largest commercial health insurance carriers in the U.S. [27], while Moorman et al. focused on 8,810 patients receiving care within four U.S. health systems [29]. The proportion of chronic HCV-infected patients prescribed pegylated interferon plus ribavirin ranged from 36–38%, with a pooled proportion of 36.7% (95% CI, 35.8–37.6%; I^2^ = 4%). Thus, 555,883 persons (15.9% [95% CI, 15.5–16.3%] of 3.5 million) are estimated to be aware of their chronic HCV diagnosis, have access to healthcare, and have received HCV therapy (cascade step 6). This estimate differed from data reported by the VA system, where 17.9% (6,224 of 34,749) of chronic HCV-infected patients have been prescribed pegylated interferon plus ribavirin treatment [26]. Using these VA-based data, 271,125 people (7.7% [95% CI, 7.6–7.9%] of 3.5 million) are estimated to be aware of their chronic HCV diagnosis, have access to healthcare, and received HCV treatment (cascade step 6).

Three non-VA studies examined SVR rates among chronic HCV-infected patients who received antiviral therapy [23], [28], [30]. These included an analysis of 575 patients (71% HCV genotype 1, 29% HCV genotype 2/3) within 40 managed care plans [28]; a study of 407 patients (57% HCV genotype 1, 42% HCV genotype 2/3) in care within a network composed of 21 community clinics and one academic health center [23]; and an evaluation of 258 patients in an integrated health care system (55% HCV genotype 1/4/6, 45% HCV genotype 2/3) [30]. The proportion of treated chronic HCV-infected patients who achieved SVR varied by genotype, with a pooled proportion of 47.0% (95% CI, 42.0–52.1%; I^2^ = 0%) for genotype 1, 75.1% (95% CI, 65.3–84.9%; I^2^ = 74%) for genotype 2/3, and 58.8% (95% CI, 56.1–61.5%; I^2^ = 0%) for all genotypes. Using the overall pooled proportion, 326,859 people (9.3% [95% CI, 8.9–9.8%] of 3.5 million) are estimated to be aware of their chronic HCV diagnosis, have access to healthcare, have been prescribed antiviral therapy, and achieved SVR (cascade step 7). In comparison, a national study of 16,864 chronic HCV-infected Veterans who received antiviral therapy (72% HCV genotype 1, 28% HCV genotype 2/3) reports an overall SVR rate of 44.1% [24]. Using these VA-based data, 245,144 people (7.0% [95% CI, 6.9–7.1%] of 3.5 million) are estimated to be aware of their chronic HCV diagnosis, have access to healthcare, received HCV treatment (based on the VA estimate reported above), and achieved SVR (cascade step 7).

## Discussion

This review identifies large gaps between current practice and treatment goals for people with chronic HCV infection. It also highlights multiple opportunities for improving engagement along the HCV treatment cascade, particularly in the diagnosis and awareness of infection, prescription of antiviral therapy, and achievement of SVR. Our study confirms findings reported in a commentary by Holmberg *et al.* that only 5–6% of all people with chronic HCV infection in the U.S. successfully progressed from detection of HCV infection to achievement of SVR [16] and extends this work by rigorously reviewing and meta-analyzing the literature, highlighting differences between U.S. Veteran and non-Veteran populations. In addition, the proposed HCV treatment cascade provides a framework for evaluating the delivery of HCV care over time and within subgroups, which will be necessary to monitor the impact of new screening efforts [33], [34] and advances in antiviral therapy [34], [35] which will likely increase the number of people completing each step along the cascade.

In 2012, the Centers for Disease Control and Prevention recommended one-time HCV testing without prior ascertainment of risk for persons born between 1945 and 1965 - a group estimated to account for three-fourths of all HCV infections in the U.S. [34]. Implementation of this recommendation, which also calls for referral of newly identified HCV-infected persons for management, may improve the proportion of individuals diagnosed and aware of their infection and referred to care.

Hepatic fibrosis assessment via liver biopsy is not required for HCV therapy, as per current professional guidelines [11]. However, staging of liver fibrosis remains important for determining the urgency and timing of HCV therapy and for identification of cirrhosis, which should prompt screening for hepatocellular carcinoma and monitoring for the development of hepatic decompensation [36], [37]. We therefore included hepatic fibrosis assessment as a step in the HCV treatment cascade. While liver biopsy has traditionally been the “gold standard” for assessing liver disease, noninvasive tests, including serum biochemical markers (e.g., aspartate aminotransferase-to-platelet ratio index, FIB-4, HCV FibroSure [LabCorp, Burlington, NC], Hepascore [San Juan Capistrano, CA], Fibrotest [Biopredictive, Paris, France]) and imaging modalities (e.g., transient elastometry [FibroScan, Echosens, Paris, France]), are increasingly playing a role in staging liver fibrosis screening [11], [33], [38]. As these noninvasive and more convenient tests evolve, it is likely that the number of patients undergoing hepatic fibrosis staging will increase.

The advent of new direct-acting antiviral agents will shorten treatment duration, likely increase the number of people offered treatment, and improve HCV cure rates (final two steps of the HCV treatment cascade) [15], [35]. However, educating providers and the general public about HCV prevention, care, and treatment; ensuring access to providers skilled in the treatment of HCV infection; and addressing the high cost of these agents will be critical to maximizing the benefits of these new therapies [14], [39]. In a recent cost-effectiveness simulation evaluating birth-cohort HCV screening and subsequent treatment of HCV-infected adults, Rein and colleagues note that birth-cohort screening followed by HCV treatment including direct-acting antiviral agents will increase quality-adjusted life-years (QALYs) by $532,200 and medical costs by $19.0 billion, for an incremental cost-effectiveness ratio of $35,700 per QALY saved (95% credible interval, $28,200 to $47,200) [40]. While these simulations accounted for common HCV-associated complications, funders, public health administrators, and providers should be aware of the financial burden of untreated HCV infection. Using data from the 2010 U.S. National Health and Wellness Survey, El Khoury et al. note that persons with untreated HCV infection had significantly (p<0.001) higher annual productivity losses ($10,316 vs. $5,459 per employed person) and annual all-cause healthcare costs ($22,818 vs. $15,362 per person) compared to HCV-uninfected individuals. [41] Evaluating the trade-off between the benefits and costs of these new agents will be critical to scaling up HCV treatment [35].

Individuals' progression along the HCV treatment cascade varied widely, with large drop-offs occurring at diagnosis and awareness of infection, prescription of antiviral therapy, and achievement of SVR. Studies are needed to evaluate and compare the population-level benefits and costs of interventions, such as screening and treatment efforts, at each step. These data can further assist public health officials in allocating resources to increase the proportion of chronic HCV-infected patients completing the cascade steps. In addition, monitoring the HCV treatment cascade over time will be critically important to defining overall progress and identifying persistent barriers at individual steps.

Since the VA system is the largest single provider of HCV care in the U.S. and because of potential differences in the management of chronic HCV infection among U.S. Veterans [42], we conducted a separate analysis to estimate the numbers of chronic HCV-infected people completing steps 5–7 in the HCV treatment cascade using VA-specific data. Among chronic HCV-infected Veterans in care, the proportion of those who received hepatic fibrosis staging by liver biopsy and who were prescribed HCV treatment was 22% and 19% lower, respectively, compared to the general population. Similarly, among Veterans with chronic HCV infection who were prescribed pegylated interferon plus ribavirin, a smaller proportion achieved SVR compared to the general population (44% vs. 58%). These variations may be due to differences in patient populations or in VA provider practices. Prior studies indicate that Veterans with chronic HCV have higher rates of absolute and relative contraindications to HCV treatment than persons seen at non-VA clinics [43]–[45]. In addition, differences in medication adherence [12], provider management, and health system factors may contribute to the lower proportion of Veterans completing the final steps of the HCV treatment cascade. Future studies should evaluate how the HCV treatment cascade changes for Veterans and non-Veterans with the advancement of new direct-acting antiviral therapies.

This study has several potential limitations. First, our treatment cascade included seven steps, as identified by prior research [8], [9], evaluated at one time point. Future studies should explore the addition (e.g. linkage to and retention in HCV care) and deletion (e.g., liver biopsy) of cascade steps to best assist public health officials in monitoring HCV care in the U.S. Further, since patients receive care over time, the studies included in this analysis may not have allowed sufficient time for certain steps in the HCV treatment cascade to take place. Monitoring the steps of the cascade over time may help to define overall progress toward population-based goals and barriers at individual steps. Second, our systematic review was limited by the relatively small number of studies identified, particularly for cascade steps 1–3. Many studies excluded prisoners and homeless individuals, populations heavily affect by chronic HCV infection. These exclusions may underestimate cascade steps, particularly access to outpatient care. Third, we broadly focused on estimating the number of chronic HCV-infected people in the general population. Estimates for each step in the HCV treatment cascade could not be determined by sex, race/ethnicity, socioeconomic status, injection drug use, and HIV status because these data were not available in the included studies. Future studies should determine the cascade within these subgroups. Fourth, HCV RNA confirmation in evaluated studies was restricted to patients' healthcare network. This may underestimate HCV RNA testing, particularly for patients who transfer care between providers. Similarly, liver biopsy was used as the marker for HCV disease staging; however, this may underestimate the number of people who were staged through the use of noninvasive tests. Fifth, not all patients immediately require or are eligible to receive antiviral therapy for chronic HCV, which may underestimate the proportion of patients treated. Further, failure to achieve SVR may be a consequence of biological factors, including HCV genotype, treatment efficacy and tolerability, or adherence to therapy. Differentiating between these causes may be important because each requires a different intervention strategy. In addition, data on prescription and SVR rates of newer direct-acting anti-HCV medications were not available. Monitoring how these steps change as providers increasingly use these new therapies will be critical. Lastly, we present the treatment cascade for people with chronic HCV infection living in the U.S., but evaluating the treatment cascade in other countries will be important and may help identify models for improving HCV monitoring and public health in those areas.

In summary, our results suggest that continued efforts are needed to improve HCV care in the U.S. In a field that is changing rapidly, with increased attention on HCV screening and approval of new, effective direct-acting antiviral agents, this proposed HCV treatment cascade provides a framework for identifying gaps in care. This framework will be useful in monitoring the impact of new public health initiatives, care models, and treatments. Only by increasing the number of persons completing each step in the cascade can the goals of the U.S. *Action Plan for the Prevention, Care, and Treatment of Viral Hepatitis* be achieved.

## Supporting Information

Table S1 **Search strategy to identify studies addressing the 7 HCV treatment cascade steps.**
(DOC)

Checklist S1 **PRISMA Checklist.**
(DOC)
