# Supplementary material for: The Treatment Cascade for Chronic Hepatitis C Virus Infection in the United States: A Systematic Review and Meta-Analysis
Source: PLoS One. 2014 Jul 2;9(7):e101554. doi: 10.1371/journal.pone.0101554 (PMC4079454; doi:10.1371/journal.pone.0101554)
Supplement: Table S1 — Search strategy to identify studies addressing the 7 HCV treatment cascade steps. (DOC) [file pone.0101554.s001.doc]

**Table S1.** Search strategy to identify studies addressing the 7 HCV treatment cascade steps.

| **Hepatitis C Virus** | **United States** | **Step 1** |
| --- | --- | --- |
| 1. Hepatitis C  2. Hepatitis C Infection  3. Hepatitis C Virus  4. HCV  5. HCV Infection  6. Or/1-5 | 1. United States  2. US  3. USA  4. Americ*  5. Or/1-4 | 1. Prevalence  2. Seroprevalance  3. Seroepidemiologic Studies  4. Incidence  5. Identification  6. Determination  7. Screening  8. Mass Screening  9. Hepatitis C Antibodies  10. Hepatitis C Antibody  Positive  11. Or/1-10 |

| **Step 2** | **Step 3** | **Step 4** |
| --- | --- | --- |
| 1. Awareness  2. Knowledge  3. Understanding  4. Comprehension  5. Health Knowledge,  Attitudes, Practice  6. Or/1-5 | 1. Linkage  2. Retention  3. Engagement  4. Continuity of Patient Care  5. In care  6. Outpatient Care  7. Primary Care  8. Primary Health Care  9. Or/1-8 | 1. RNA  2. RNA Positive  3. RNA, Viral  4. Viral Load  5. Viral Load Positive  6. PCR Positive  7. Nucleic Acid Testing  8. Chronic Infection  9. Hepatitis C, Chronic  10. Or/1-9 |

| **Step 5** | **Step 6** | **Step 7** |
| --- | --- | --- |
| 1. Staging  2. Liver Biopsy  3. FIB-4  4. APRI  5. Genotyping  6. Or/1-5 | 1. Therapeutics  2. Treatment  2. Therapy  3. Medication Therapy  Management  4. Drug Therapy  5. Drug Therapy, Combination  6. Antivirals  7. Antiviral Agents  8. Regimen  9. Prescription  10. Prescriptions  11. Or/1-10 | 1. Sustained Virologic  Response  2. SVR  3. Suppression  4. Treatment Success  5. Cure  6. Treatment outcome  7. Or/1-6 |

Footnote: For each question, searches include terms for that question as well as those for Hepatitis C Virus and United States
